# Disparities in Spatial Prevalence of Feline Retroviruses due to Data Aggregation: A Case of the Modifiable Areal Unit Problem

**DOI:** 10.1155/2014/424138

**Published:** 2014-02-19

**Authors:** Bimal K. Chhetri, Olaf Berke, David L. Pearl, Dorothee Bienzle

**Affiliations:** ^1^Department of Population Medicine, Ontario Veterinary College, University of Guelph, Guelph, ON, Canada N1G 2W1; ^2^Department of Mathematics and Statistics, University of Guelph, Guelph, ON, Canada N1G 2W1; ^3^Institute of Biometry, Epidemiology and Information Processing, University of Veterinary Medicine Hannover (Foundation), 30173 Hannover, Germany; ^4^Department of Pathobiology, Ontario Veterinary College, University of Guelph, Guelph, ON, Canada N1G 2W1

## Abstract

The knowledge of the spatial distribution feline immunodeficiency virus and feline leukemia virus infections, which are untreatable, can inform on their risk factors and high-risk areas to enhance control. However, when spatial analysis involves aggregated spatial data, results may be influenced by the spatial scale of aggregation, an effect known as the modifiable areal unit problem (MAUP). In this study, area level risk factors for both infections in 28,914 cats tested with ELISA were investigated by multivariable spatial Poisson regression models along with MAUP effect on spatial clustering and cluster detection (for postal codes, counties, and states) by Moran's *I* test and spatial scan test, respectively. The study results indicate that the significance and magnitude of the association of risk factors with both infections varied with aggregation scale. Further more, Moran's *I* test only identified spatial clustering at postal code and county levels of aggregation. Similarly, the spatial scan test indicated that the number, size, and location of clusters varied over aggregation scales. In conclusion, the association between infection and area was influenced by the choice of spatial scale and indicates the importance of study design and data analysis with respect to specific research questions.

## 1. Introduction

Infections with feline immunodeficiency virus (FIV) and feline leukemia virus (FeLV) have been reported from a number of countries and are important conditions in cats [1]. The most common mode of transmission of these immunosuppressive retroviruses is through bite wounds. FeLV infection is also commonly acquired via the oronasal route through mutual grooming, nursing, or sharing of dishes [2]. The known risk factors for acquiring these infections are male sex, adulthood, and exposure to outdoors, whereas being neutered and indoor lifestyle are known protective factors [3]. Recent studies estimate a seroprevalence of 2.3% (FeLV) and 2.5% (FIV) in the United States (US) [4] and 3.4% (FeLV) and 4.3% (FIV) in Canada [1].

Despite decades of discovery, clinical management of cats infected with FIV and FeLV is still challenging without the existence of an effective therapeutic intervention [3]. Therefore, better ways to control the infections and prophylactic management is the mainstay of disease prevention strategy for these infections. A number of previous studies have suggested that the prevalence of retroviral infections in domestic cat populations varies by regions and maybe attributed to variable population density, reproductive status, age, gender, and housing conditions [5–11]. For the US and Canada, spatial variation in FIV and FeLV seroprevalence has been reported in previous studies that generated data for this research [1, 4]. Here we attempt to extend the findings by applying spatial statistical methods to illustrate geographic variation in the distribution of FIV and FeLV infections and assess the relationship with group-level risk factors. Spatial epidemiological methods are commonly used to identify, describe, and quantify spatial patterns in the distribution of health events. Spatial patterns commonly of interest include trends, clustering, and detection of clusters in the occurrence of health events in a population. Furthermore, geographic correlation studies can be important tools to evaluate the association of spatial or environmental risk factors with the occurrence of health events after adjusting for confounders. The identification of such spatial patterns may provide clues for further testable hypotheses about an unknown disease etiology [12]. Ecological studies, such as geographic correlation studies, are particularly valuable when an individual level association between infection and risk factors is evident and a group-level association is assessed to determine the population health impact [13]. To this effect, spatial analysis of FIV and FeLV infections can be a valuable tool in epidemiological understanding of these infections.

Due to lack of individual level data and client confidentiality and to create meaningful units for data analysis, aggregated or area level data may be used to carry out such spatial epidemiological studies. However, the way areal units are defined can influence the results and inferences based on aggregated data. Specifically, the number or size of areas used and how the area boundaries are drawn can influence spatial data analysis. This has been termed the modifiable areal unit problem (MAUP) and is a long known phenomenon [14, 15] in the geographical literature. The MAUP stems from the fact that areal units are usually arbitrarily determined and can be modified to form units of different sizes or spatial arrangements [16]. The MAUP consists of two interrelated components—the scale and zoning effects. The scale effect is the variation in results obtained when the areal data comprising smaller areal units is grouped to form increasingly larger units. The zoning effect, on the other hand, is the variation in results obtained due to alternative formations of areal units where the number of areal units is constant, that is, analysis comprising the same number of areal units but different area shapes [14, 17, 18].

The goal of this study was to evaluate the association of seroprevalence of FIV and FeLV with ecological risk factors in a spatial regression model. Specific objectives of the study were to examine the MAUP effects on (a) the spatial clustering of FIV and FeLV infections; (b) the occurrence of high-risk clusters of FIV and FeLV infections; and (c) the relationship between area level seroprevalence and risk factors in context of aggregated covariates.

## 2. Materials and Methods

### 2.1. Data Source, Study Areas and Population

A dataset consisting of diagnostic test results from 29,182 cats tested for FIV and FeLV between August and November of the years 2004 and 2007 from the US and Canada was obtained from previous cross-sectional studies [1, 4]. The cats included in this study were conveniently sampled from veterinary clinics and animal shelters across 40 contiguous states of the US and 9 Canadian provinces encompassing 641 US zip codes and Canadian forward sortation areas in 346 US counties and Canadian census divisions [19]. The testing for FIV and FeLV was carried out in house or in laboratory employing a commercially available ELISA (SNAP Combo FeLV antigen/FIV antibody, PetCheck FIV Antibody, and PetCheck FeLV Antigen; IDEXX Laboratories) using blood, serum, or plasma. Information on postal code of testing facility, type of testing facility (clinic or shelter), age of the cat (juvenile <6 months or adult), sex, and neuter status (sexually intact female, spayed female, sexually intact male, or castrated male), access to outdoors (indoors or outdoors), and general health at time of testing (healthy or sick) was also retrieved from the dataset (Table 1(a)).

### 2.2. Data Aggregation

The three spatial aggregation scales of interest in this study were postal codes, counties and states. The US five-digit zip code and Canadian forward sortation areas (FSA) were designated as postal codes, StatCan (Statistics Canada) census divisions (CDs) were defined as corresponding to US counties, and Canadian provinces were defined as states. The counts of positive test results and number of tests for each area were aggregated to these three spatial aggregation scales of interest (641 postal codes, 346 counties, and 49 states). In addition, group-level risk factors, constructed from individual risk factors, included the proportion of juvenile cats (<6 months), intact males, intact females, cats that were exclusively indoors, cats tested at clinics, cats that were healthy at the time of testing, and the seroprevalence of FIV and FeLV. These covariates were “constructed” for respective scales using categories of individual data presented in Table 1(a).

### 2.3. Geocoding

In order to spatially reference the postal codes, counties, and states, the geographic coordinates (as centroids) of the US zip codes, counties, states, and the Canadian FSAs were obtained from Environmental System Research Institute (ESRI) postal code shapefiles [20].

Each Canadian FSA was assigned to the respective county and state based on the postal code conversion file (PCCF) available from StatCan.

### 2.4. Statistical Methods

#### 2.4.1. Spatial Clustering

To investigate disease clustering (i.e., the presence of spatial autocorrelation in the data), Moran's *I* test was applied. Given the infectious nature of FIV and FeLV, clustering was assumed to be present. The interest in this study was to evaluate whether aggregation of data from postal code level (where the data was collected) to county and states had any effect on strength and presence of clustering. In this regard, the presence and strength of spatial clustering of FIV and FeLV infections for each level of aggregation were assessed by Moran's *I* test on the smoothed seroprevalence estimates using empirical Bayesian smoothing [21]. Since the number of tested cats varied among the areas, smoothed seroprevalence estimates were estimated from crude seroprevalence (number of cats testing positive/number of cats tested) for each area using the empirical Bayesian (EB) estimation such that the area specific seroprevalence estimates were adjusted towards the overall mean. The EB estimation technique can be interpreted as internal standardization [22].

The null hypothesis of Moran's *I* test states that there is no spatial autocorrelation of FIV or FeLV seroprevalence between areas, and the respective Moran's *I* coefficient summarizes the degree to which similar observations (i.e., seroprevalence of FIV or FeLV) tend to occur near each other [17]. The Moran's *I* coefficient was estimated as follows:

(1) I=n∑i=1n∑i=jnwij(yi−y¯)(yj−y¯)(∑i=1n(yi−y¯)2)(∑∑i≠jwij),

where  *n*: number of areas,  *w*_*ij*_: measure of spatial proximity between areas *i* and *j*,  *y*_*i*_: Poisson model based EB smoothed seroprevalence of FIV or FeLV in area *i*,  *y*_*j*_: Poisson model based EB smoothed seroprevalence of FIV or FeLV in area *j*, and  $\bar{y}$: overall EB smoothed seroprevalence.

*w*_*ij*_ is the spatial weights matrix which considers three nearest neighbours (*w*_*ij*_ is 1 if area *i* and *j* are within a distance of three nearest neighbours and zero if otherwise). The Moran's *I* test was applied using the spdep package of statistical software *R* [23].

#### 2.4.2. Spatial Cluster Detection

While Moran's *I* summarizes the overall clustering pattern in the study area, disease cluster detection methods are used to identify the locations of clusters and thus are location specific. Of various methods proposed for cluster detection [17], the most widely used is the spatial scan test [24]. Here, the MAUP effect on the spatial scan test was investigated with respect to FIV and FeLV infections. Furthermore, the spatial scan test can be extended to detect clusters after adjustment for known risk factors or confounders for FIV and FeLV infections. Therefore, the presence of statistically significant high-risk clusters of FIV (or FeLV) infection was investigated using a spatial scan test adjusted for risk factors under the Poisson assumption [25], as implemented in SaTScan version 9.0 [26].

The spatial scan test identifies potential clusters using circular windows of varying radius (size) and location (area centroids) across the study area. To apply the Poisson model, it was assumed under the null hypothesis that the number of FIV or FeLV cases in each area followed Poisson distribution with the expected number of cases in each area proportional to the covariate (risk factor) adjusted tested cat population [27]. High-risk cluster detection was performed by comparing the observed number of cases within the scanning window with the expected number, that is, if cases were to be distributed randomly in space [25]. In other words, detection of high-risk clusters would indicate the prevalence of FIV (or FeLV) inside the circular window as significantly higher than outside the window. The statistical significance of the clusters was established by Monte Carlo hypothesis testing using 999 Monte Carlo replications with a significance level set to *α* = 5%. The significance of multiple clusters was tested sequentially conditional on the presence of the previously detected clusters such that secondary clusters were tested and reported only if the more likely clusters were significant [28]. The size of the scanning window in the spatial scan statistic was allowed to increase from individual areas and expanded to include neighbouring areas until a maximum of 50% of the total tested population. No geographical overlap of clusters was allowed. Detected clusters were visualized by plotting respective circles on a map of the study area. The characteristics of detected clusters were compared across aggregation levels to assess the MAUP effect.

#### 2.4.3. Spatial Regression Modeling

Apart from describing the spatial patterns of disease in terms of clustering and cluster, geographic correlation analysis (or spatial regression modeling) for spatial data was carried out to quantify the effect of spatially referenced group-level risk factors on the spatial distribution of disease events, that is, FIV and FeLV infections [17, 29]. While these studies are similar to ecological regression methods, it is critical to adjust for the spatial autocorrelation in the data in order to prevent type *I* errors, that is, providing “statistically significant” results when none exists [30]. Among many proposed methods for spatial regression modeling for areal data [17, 29, 31, 32], Poisson distributed counts for rare disease or infections such as FIV and FeLV can be effectively modeled to assess its relationship with group-level risk factors using generalized linear mixed models (GLMM) with spatially correlated random effects, also known as spatial GLMM. In this study, interest was to evaluate group-level risk factors for FIV and FeLV infections as well as to quantify the effect of MAUP as change in magnitude and significance of regression parameters with spatial aggregation scale. For each aggregation level, the count of FIV and FeLV infections in each area was modelled as a function of the group-level covariates in a Poisson regression model framework with the log of number of tested cats as the offset.

Prior to inclusion of covariates in the regression models, the relationship between the outcome and covariates was assessed for linearity by plotting the log of the seroprevalence of infection for both FIV and FeLV against the covariate using a locally weighted regression. The covariates were modelled as dichotomized variables if the relationship was deemed to be nonlinear. This decision was taken to ensure comparability of covariates across the aggregation levels. Covariates were modeled as dichotomous variables with cut-offs for low and high categories set at median value (50%) of the respective covariates. When modeled as predictor variable and not the outcome, the cut-off for categories of covariate FIV and FeLV seroprevalence was set at 3%, 8% and over 8%. The cut-off of 3% is the general prevalence of FIV and FeLV in cats in North America. Since all the covariates are deemed clinically important risk factors, they were included as fixed effects in a multivariable model, with no interactions. Further, the same model was fit to data at all 3 levels of aggregation (state, county, and postal code) to avoid any influence of the selection method or covariate(s) exclusion in the comparison of models [33]. For state level aggregation, covariates with sample size less than five were omitted.

In order to account for spatial autocorrelation and overdispersion in the models, an exponential spatial covariance structure was introduced and the models were rerun using penalized quasilikelihood (PQL) estimation [32, 34]. An exponential covariance structure was based on a semivariogram fitted to the deviance residuals of the Poisson regression models and was deemed biologically appropriate because, for infectious agents such as FIV and FeLV, areas in proximity are expected to be similar with respect to disease prevalence.

The presence of overdispersion in (nonspatial) Poisson regression models was evaluated by testing the model deviance against degrees of freedom using a *χ* ^2^ distribution and a 5% significance level [35]. Multicollinearity was tested among the covariates in the multivariable model by estimating the variance inflation factor (VIF), and all variables with a VIF value of 10 or above were considered collinear [36]. All statistical modeling was done using statistical software *R* [37].

## 3. Results and Discussion

### 3.1. Results

#### 3.1.1. Descriptive Statistics

A total of 28,914 test results were included in this study from 688 veterinary clinics and 158 animal shelters from 40 states of the US and 9 Canadian provinces encompassing 346 counties and 641 postal codes. A total of 634 recorded postal codes (out of 648) were accurately matched during geocoding. Seven records were reassigned to proper postal codes using clinic address. In total, geographic coordinates were retrieved for 641 postal codes (out of 648) for 28,914 cats (out of 29,182 cats).

The individual characteristics of FIV and FeLV infected cats and descriptive statistics of area wise counts are presented in Tables I(a) and I(b). Overall the observed seroprevalence of FIV was higher than that of FeLV, 3.16% and 2.71%, respectively. The mean and variability in number of cats with positive test results for both infections and the number of cats tested increased with higher level of aggregation but decreased for seroprevalence (Table 1(b)). The seroprevalence of FIV infection for postal codes, counties and states ranged from 0–100%, 0–50%, and 0–13% respectively, while the seroprevalence of FeLV ranged from 0–100%, 0–33%, and 0–20% for postal code, county, and state levels, respectively.

#### 3.1.2. Spatial Clustering

The results of Moran's *I* clustering test on EB smoothed seroprevalence is presented in Table 2. Moran's *I* statistic indicated significant spatial clustering in seroprevalence of infection for FIV at postal code and county level aggregations (*I* = 0.09 and *I* = 0.15 resp., *P* < 0.01), Likewise, spatial clustering was identified for FeLV at postal code and county level aggregations (*I* = 0.12 and 0.15, resp., *P* < 0.01). At state level of aggregation no spatial clustering was detected.

#### 3.1.3. Spatial Cluster Detection

**Figure 1:**
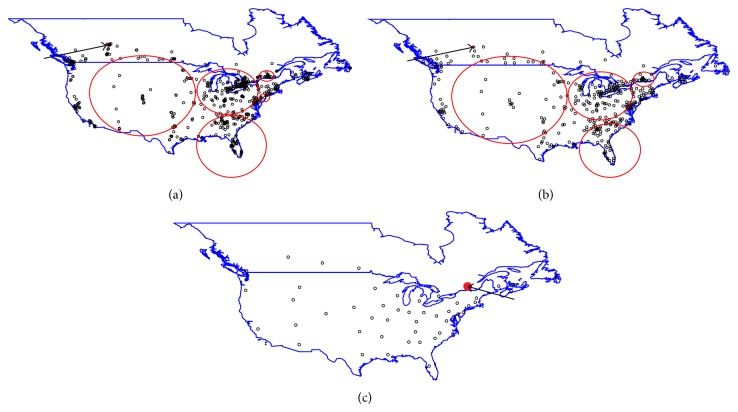
Spatial clusters of FIV infections (red circles) identified by the spatial scan test at postal code, county, and state level aggregations. Arrows indicate clusters hidden by the black open circles that represent region centroids. (a) Clusters at postal code level aggregation. (b) Clusters at county level aggregation. (c) Cluster at state level aggregation.

**Figure 2:**
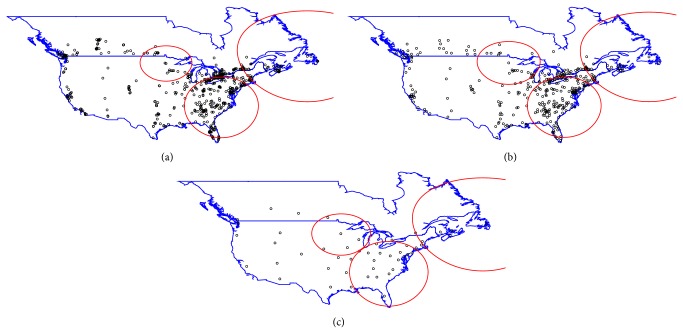
Spatial clusters of FeLV infections (red circles) identified by the spatial scan test at postal code, county and state level aggregations. Black open circles represent region centroids. (a) Clusters at postal code level aggregation (b) Clusters at county level aggregation (c) Cluster at state level aggregation.

Tables 3(a)-3(b) and Figures 1 and 2 display detailed information for all clusters identified by the spatial scan statistic. For both FIV and FeLV infections, spatial clusters were detected at all aggregation levels. However, the numbers of clusters detected for FIV and FeLV infections varied with the level of aggregation. For FIV infections, one cluster was detected for state, five for county, and six for postal code level aggregations. Some clusters identified at postal code level were not detected at county level and state level aggregations (Table 3(a) and Figures 1(a)–1(c)). For FeLV, three clusters each for state, county, and postal code levels were identified, with location and size of the clusters slightly varying by spatial scale (Table 3(b) and Figures 2(a)–2(c)). Figures 2(a)–2(c) indicate that FeLV clusters were about the same size and in the same location for postal code and county levels of aggregation, whereas clusters at the state level differed with respect to size and, more importantly, location.

#### 3.1.4. Spatial Regression Modeling

Spatial Poisson regression indicated that the seroprevalence of FeLV infections was observed to be lower among areas with greater proportion of cats that were young and indoors (Table 4(a)). Conversely, seroprevalence of FeLV infections was higher among areas with a greater proportion of intact males and cats tested at clinics and with a higher seroprevalence of FIV (Table 4(a)). Similarly, seroprevalence of FIV infection was higher among areas with a greater proportion of cats tested at clinics and with a higher seroprevalence of FeLV (Table 4(b)). The seroprevalence of FIV, however, was lower in areas with greater proportion of intact females. The significance and magnitude of observed associations were not consistent across all aggregation levels. The direction of change in magnitude of association was also not consistent. Associations seen at postal code and county levels may not be evident at the state level (e.g., percentage of juvenile cats in an area and FeLV). Or conversely, associations observed at state level were not detected at lower levels (e.g., percentage of male intact in an area and FeLV).

### 3.2. Discussion

This study showed that commonly used spatial epidemiological methods (Moran's *I*, spatial scan test, and spatial regression modeling) are sensitive to choice of the spatial aggregation scale for analysis, that is, affected by the MAUP. Recognizing the importance of bias due to the MAUP is important for the validity of spatial epidemiological inferences.

Moran's *I* coefficient indicated clustering of FIV and FeLV positive test results. However, the strength and significance of clustering varied across spatial aggregation levels. Given the infectious nature of both retroviruses, areas near each other are expected to have similar seroprevalence levels. Therefore, positive autocorrelation in FIV and FeLV seroprevalence was expected. As the data are aggregated, variations at lower levels of aggregation dissolve to form more homogenous areas in terms of population characteristics [17]. With the postal codes aggregated to counties and states, the variability in seroprevalence estimates evident at the scale of postal code and counties likely diminished as the seroprevalence estimates were averaged (Table 1(b)). Generally, spatial aggregation is expected to increase spatial correlations [38]. However, in this study the opposite effect was observed, and the spatial autocorrelation present at postal and county levels disappeared at state level. This may imply that the biological processes which are associated with the clustering of infected cats at local levels (i.e., postal codes and counties) become irrelevant or unobservable at higher aggregation levels (i.e., states). It is important to note that there is a random aspect to the effects of the MAUP and it may be difficult to generalize about how different datasets with different spatial units are affected by the MAUP [15]. Furthermore, the aggregation process itself may induce positive spatial autocorrelation, particularly if the aggregation process allows overlapping units [15] such as postal code to form counties. Unfortunately, not all postal code areas or counties in the US and Canada were sampled for this study and Moran's *I* test was based on a neighbourhood specification of three nearest neighbours. Therefore it is possible to get “first three nearest neighbours” areas too distant from a biological perspective on infection, which would tend to aggravate variability and reduce autocorrelation at lower levels of aggregation.

Evidence of high-risk areas for FIV and FeLV infections as detected by the spatial scan test adjusted for known confounders suggests that yet unknown spatial factors may exist. This study also indicates that the results from the spatial scan test can be influenced by spatial aggregation as evident from the difference in size, number, and location of clusters for both FIV and FeLV infections.

Despite differences in cluster characteristics with respect to size and location across aggregation levels for FeLV, no clusters were detected in the western parts of United States and Canada indicating that these areas had lower prevalence of infection compared to the rest of the study area. The results at county and postal code area levels were similar with respect to cluster size and locations. The sampled counties and postal codes were not very different with respect to population characteristics and thus may be insensitive to aggregation effects. However, it is most likely an artificial effect as most counties had only a few postal codes sampled within them. While multiple clusters were detected for FIV at the postal code level, these were not detected at higher aggregation levels (Figures 1(a)–1(c)). Spatial aggregation reduced the sample size from 641 postal codes to 346 counties and 49 states (or provinces) in this study. Aggregation of data may smooth out local effects but may also lead to reduced power to detect small clusters while stabilizing rates that may be unstable in smaller areas due to smaller at-risk-populations in the denominator [39, 40].

The results from spatial Poisson regression modeling indicate that the seroprevalence of both infections is higher in areas with the greater proportion of cats tested at clinics than at shelters. Although the seroprevalence of FIV and FeLV in shelter cat populations mirrors that of cat populations served by veterinary clinics [3], the reasons for testing may be different. Testing in shelters is driven by housing considerations and potential for adoption, whereas at clinics mostly sick cats are tested [41]. Thus, the seroprevalence estimates in populations tested at clinics may be inflated.

An increase in seroprevalence of FeLV was found to be associated with higher FIV seroprevalence. This is expected since both infections share similar risk factors [42] and as a result would have similar infection rates. Furthermore, the seroprevalence of FeLV in an area was negatively associated with a higher proportion of young cats, indoor cats, and neutered males. Consequently, the seroprevalence would be higher in areas with greater proportions of adults, outdoor cats, and intact cats due to social interactions related to roaming, breeding, and fighting. Therefore, the areas populated with cats of these characteristics can be expected to harbour cats with higher risk of acquiring retroviral infections. Areas with greater proportions of intact female cats had a lower seroprevalence for FIV than areas with greater proportions of spayed female cats. This finding seems to be counterintuitive from a biological perspective, as similar to areas with greater proportions of intact males; populations with greater proportions of intact females might be expected to be more susceptible to acquire an infection as a result of higher probabilities of animals fighting. However, the predictors that are derived variables (variables constructed as summaries of individual characteristics) in the group level analysis cannot distinguish the individual-level effect of the variable from its contextual or group level effect [43]. Derived variables are constructed mathematically by summarizing the individual characteristics in a group [43], for example, proportion of males in an area.

The significance and magnitude of associations between health status and risk factors (or predictor variables) are governed by the scale of spatial aggregation. The associations observed at one scale should be used with caution when inferences are made at another scale. Except for FIV and FeLV seroprevalence, this study did not identify any covariate consistently associated with the outcome across all three aggregation levels. The geographic scales on which these two variables are meaningful factors probably include scales larger than postal code. This is likely true, since veterinary clinics generally service areas that overlap several postal code areas or occasionally across county barriers. For other variables, the choice of the aggregation scale seems to affect the significance and magnitude of observed associations. Generally, most of the predictor variables were only significant at lower levels of aggregation. Suggesting that seroprevalence of FIV and FeLV at higher levels of aggregation depend on further group-level factors not considered in this study. It is important that this spatial scale dependence is not overinterpreted as a sole MAUP effect as multivariable analysis is a complex subject and nevertheless can be prone to missing but confounding variables [16]. This study utilized an ecological regression framework based on covariates as derived variables from individual level data [44]. Thus, the associations observed between covariates and the seroprevalence pertain to group levels. It is necessary to be cautious in extrapolating these findings to the individual level due to the potential for ecological bias.

Currently, there are no solutions to fully overcome the effects of MAUP and related methodological issues have not yet been adequately addressed. Recommendations have been made to minimize MAUP effects in statistical inference by analyzing the aggregated covariates in hierarchical levels of areal units from the finest spatial resolution possible to a coarser resolution, verifying consistent model results across different scales, avoiding ecological fallacy, collecting data at the scale at which inferences is to be made, and using scale invariant statistics to make inferences [17, 44–46].

## 4. Conclusion 

This study demonstrated the importance of study design in the context of spatial epidemiological studies. Inference from spatial epidemiological studies dealing with aggregated data could potentially be affected by the modifiable areal unit problem (MAUP). The MAUP can result in overlooking or conversely overstating the effect of risk factors and influence statistics designed to test for clustering and clusters. In the present study of FIV and FeLV seroprevalence among cats across the US and Canada it was found that disease clusters may become unidentifiable when data are aggregated. Therefore, it is of utmost importance that investigators define the appropriate scale for data collection and analysis with respect to their research questions.

## Figures and Tables

**Table:** (a)

Factors	Tested^a^	FIV positive	Prevalence (95% CI)^b^	FeLV positive	Prevalence (95% CI)^b^
Testing site					
Veterinary clinic	19314	674	3.5 (3.2–3.8)	617	3.2 (2.9–3.4)
Shelter	9600	241	2.5 (2.2–2.8)	166	1.7 (1.5–2.0)
Age					
Juvenile	15461	160	1.0 (0.9–1.2)	198	1.3 ( 1.1–1.5)
Adult	13453	755	5.6 (5.2–6.0)	585	4.4 (4.0–4.7)
Sex					
Male intact	8649	372	4.3 (3.9–4.8)	240	2.8 (2.4–3.1)
Male castrated	6027	299	5.0 (4.4–5.5)	198	3.3 (2.9–3.8)
Female intact	9211	139	1.5 (1.3–1.8)	198	2.2 (1.9– 2.5)
Female spayed	4987	102	2.1 (1.7–2.5)	144	2.9 (2.4–3.4)
Unknown	40	3	7.5 (1.7–20.4)	3	7.5 (1.7–20.4)
Outdoor exposure					
No	7142	99	1.4 (1.1–1.7)	136	1.9 (1.6–2.3)
Yes	17968	708	3.9 (3.7–4.2)	565	3.1 (2.9–3.4)
Unknown	3804	108	2.8 (2.3–3.4)	82	2.2 (1.7–2.7)
Health status					
Healthy	22311	507	2.3 (2.1– 2.5)	379	1.7 (1.5–1.9)
Sick	6092	389	6.4 (5.8–7.0)	391	6.4 (5.8–7.1)
Unknown	511	19	3.7 (2.3–5.7)	13	2.5 (1.4–4.3)

^a^Total number of cats tested for FIV and FeLV infection. Cats were tested at the same time for both FIV and FeLV infections.

^b^CI: confidence intervals for seroprevalence estimates with *α* = 0.05.

**Table:** (b)

Infection	Aggregation level	Characteristics^a^	Total	Mean	SD^b^	Range
FIV	State	Seroprevalence		3	2	0–13
		Cases	915	18.67	35.14	0–221
		Tested	28914	590.08	903.01	8–5732
	County	Seroprevalence		4	5	0–50
		Cases	915	2.64	5.28	0–59
		Tested	28914	83.57	125.81	1–958
	Postal code	Seroprevalence		4	7	0–100
		Cases	915	1.43	1.78	0–26
		Tested	28914	45.11	61.58	1–838

FeLV	State	Seroprevalence		3	3	0–20
		Cases	783	15.98	25.25	0–145
		Tested	28914	590.08	903.01	8–5732
	County	Seroprevalence		3	4	0–33
		Cases	783	2.26	4.24	0–47
		Tested	28914	83.57	125.81	1–958
	Postal code	Seroprevalence		3	6	0–100
		Cases	783	1.22	2.12	0–19
		Tested	28914	45.11	61.58	1–838

^a^The descriptive statistics for seroprevalence pertain to mean value among states, counties, or postal codes. For example, min. and max. seroprevalence estimates for FIV among states are 0 and 13, respectively.

^b^Standard deviation.

**Table 2:** Moran's *I* statistics based on empirical Bayesian smoothed seroprevalence of FIV and FeLV infections by spatial aggregation level.

Infection	Areal unit	*I*	*E* ^a^	Var^b^	SD^c^	*P* value
FIV	Postal code	0.09	−0.002	0.001	3.30	<0.01
	County	0.15	−0.003	0.002	3.82	<0.01
	State	−0.06	−0.021	0.409	−0.37	0.66

FeLV	Postal code	0.12	−0.002	0.001	4.05	<0.01
	County	0.15	−0.003	0.002	3.53	<0.01
	State	0.00	−0.021	0.011	0.18	0.42

^a^Expected value of Moran's *I* under the null hypothesis of no spatial autocorrelation; ^b^variance; ^c^standard Deviation.

**Table:** (a)

Cluster	Coordinates^a^	Radius^b^	Obs^c^	Pop^d^	Exp^e^	Obs/exp	*P* value
State							
1	45.894, −73.425	0.00	118	1270	72.56	1.63	<0.01
County							
1	41.615, −73.201	191.69	118	1191	69.26	1.70	<0.01
2	53.329, −114.075	0.00	33	462	13.46	2.45	<0.01
3	41.621, −83.653	641.51	345	9648	279.73	1.23	<0.01
4	28.515, −81.324	715.81	84	2545	52.83	1.59	<0.01
5	40.666, −105.461	1163.84	86	2336	52.41	1.64	<0.01
Postal code							
1	53.572, −114.046	0.00	25	25	0.70	35.54	<0.01
2	45.578, −73.800	147.14	127	1322	72.45	1.75	<0.01
3	41.650, −83.673	638.28	345	9625	274.98	1.25	<0.01
4	27.817, −82.6777	864.23	101	3085	65.73	1.54	<0.01
5	40.595, −105.129	1123.52	84	2260	49.19	1.71	<0.01
6	40.105, −74.353	109.25	22	645	8.34	2.64	<0.01

^a^Longitude and latitude coordinates of the center of cluster; ^b^radius in kilometers;^ c^observed number of ELISA positive cats; ^d^total number of cats in the cluster; ^e^expected number of ELISA positive cats under Poisson assumption.

**Table:** (b)

Cluster	Coordinates^a^	Radius^b^	Obs^c^	Pop^d^	Exp^e^	Obs/exp	*P* value
State							
1	48.045, −54.689	1437.00	164	2827	93.48	1.75	<0.01
2	45.228, −93.998	637.96	78	1918	47.37	1.65	<0.01
3	34.341, −80.767	999.14	272	10089	209.11	1.30	<0.01
County							
1	48.785, −55.986	1381.90	162	2789	90.83	1.78	<0.01
2	47.109, −94.917	660.90	81	1697	43.05	1.87	<0.01
3	34.841, −79.480	932.22	275	9791	209.66	1.31	<0.01
Postal code							
1	48.949, −55.634	1403.07	150	2337	75.66	1.98	<0.01
2	46.948, −94.824	545.70	64	1169	31.03	2.06	<0.01
3	34.767, −79.452	936.10	274	9680	206.15	1.30	<0.01

^a^Longitude and latitude coordinates of the center of cluster; ^b^radius in kilometers;^ c^observed number of ELISA positive cats; ^d^total number of cats in the cluster; ^e^expected number of ELISA positive cats under Poisson assumption.

**Table:** (a)

	Postal code	*P* value	County	*P* value	State	*P* value
	PR^a^ (95% CI)		PR^a^ (95% CI)		PR^a^ (95% CI)	
% juvenile						
≤50	Ref		Ref		Ref	
>50	0.66 (0.52–0.84)	<0.01	0.78 (0.65–0.94)	<0.01	0.74 (0.5–1.08)	0.13
% female intact						
≤50	Ref		Ref		Ref	
>50	1.25 (0.97–1.61)	0.09	1.18 (0.96–1.45)	0.12	0.63 (0.36–1.09)	0.10
% male intact						
≤50	Ref		Ref		Ref	
>50	1.05 (0.82–1.35)	0.69	0.88 (0.72–1.07)	0.19	2.06 (1.12–3.77)	<0.05
% indoors						
≤50	Ref		Ref		—	—
>50	0.62 (0.48–0.81)	<0.01	0.8 (0.63–1.02)	0.07	—	—
% healthy						
≤50	Ref		Ref		—	—
>50	0.99 (0.74–1.32)	0.93	1.07 (0.82–1.4)	0.63	—	—
% tested at clinics						
≤50	Ref		Ref		Ref	
>50	1.79 (1.34–2.39)	<0.01	1.26 (1.04–1.54)	0.02	1.29 (0.86–1.92)	0.22
FIV seroprevalence						
<3.0	Ref		Ref		Ref	
3.0–8.0	1.42 (1.12–1.80)	<0.01	1.17 (0.98–1.4)	0.08	1.11 (0.78–1.57)	0.55
>8.0	2.44 (1.80–3.33)	<0.01	2.4 (1.87–3.09)	<0.01	2.6 (1.27–5.32)	<0.05

Intercept: −4.86, −3.86, and −3.99 for postal code, county, and state levels respectively, with a *P* value of <0.01.

^a^Prevalence ratios obtained by exponentiation of respective coefficients and their 95% confidence intervals. Rate/risk ratios are interpreted as prevalence ratios.

**Table:** (b)

	Postal code	*P* value	County	*P* value	State	*P* value
	PR^a^ (95% CI)		PR^a^ (95% CI)		PR^a^ (95% CI)	
% juvenile						
≤50	Ref		Ref		Ref	
>50	0.76 (0.56–1.02)	0.07	0.91 (0.71–1.16)	0.44	0.83 (0.57–1.21)	0.35
% female intact						
≤50	Ref		Ref		Ref	
>50	0.73 (0.53–0.99)	0.04	0.77 (0.59–1)	0.05	0.94 (0.55–1.62)	0.83
% male intact						
≤50	Ref		Ref		Ref	
>50	0.98 (0.71–1.34)	0.89	1.01 (0.78–1.3)	0.96	0.88 (0.48–1.59)	0.67
% indoors						
≤50	Ref		Ref		—	—
>50	1.03 (0.72–1.48)	0.87	0.85 (0.58–1.24)	0.39	—	—
% healthy						
≤50	Ref		Ref		—	—
>50	1.08 (0.73–1.60)	0.70	0.85 (0.61–1.2)	0.36	—	—
% tested at clinics						
≤50	Ref		Ref		Ref	
>50	1.03 (0.77–1.39)	0.84	1.46 (1.13–1.89)	<0.01	1.23 (0.85–1.76)	0.28
FeLV seroprevalence						
<3.0	Ref		Ref		Ref	
3.0–8.0	1.57 (1.17–2.11)	<0.01	1.29 (1.02–1.63)	0.04	1.18 (0.82–1.69)	0.38
>8.0	2.30 (1.60–3.29)	<0.01	2.01 (1.44–2.81)	<0.01	5.19 (1.16–23.25)	0.04

Intercept: −3.40, −3.30, and −3.39 for postal code, county, and state levels respectively, with a *P* value of <0.01.

^a^Prevalence ratios obtained by exponentiation of respective coefficients and their 95% confidence intervals. Rate/risk ratios are interpreted as prevalence ratios.
